# Compensation or Preservation? Different Roles of Functional Lateralization in Speech Perception of Older Non-musicians and Musicians

**DOI:** 10.1007/s12264-024-01234-x

**Published:** 2024-06-05

**Authors:** Xinhu Jin, Lei Zhang, Guowei Wu, Xiuyi Wang, Yi Du

**Affiliations:** 1 Institute of Psychology, Chinese Academy of Sciences, Beijing, 100101 China; 2 Department of Psychology, University of Chinese Academy of Sciences, Beijing, 100049 China; 3 CAS Center for Excellence in Brain Science and Intelligence Technology, Shanghai, 200031 China; 4 Chinese Institute for Brain Research, Beijing, 102206 China

**Keywords:** Functional lateralization, Speech perception, Aging, Musical training experience

## Abstract

Musical training can counteract age-related decline in speech perception in noisy environments. However, it remains unclear whether older non-musicians and musicians rely on functional compensation or functional preservation to counteract the adverse effects of aging. This study utilized resting-state functional connectivity (FC) to investigate functional lateralization, a fundamental organization feature, in older musicians (OM), older non-musicians (ONM), and young non-musicians (YNM). Results showed that OM outperformed ONM and achieved comparable performance to YNM in speech-in-noise and speech-in-speech tasks. ONM exhibited reduced lateralization than YNM in lateralization index (LI) of intrahemispheric FC (LI_intra) in the cingulo-opercular network (CON) and LI of interhemispheric heterotopic FC (LI_he) in the language network (LAN). Conversely, OM showed higher neural alignment to YNM (i.e., a more similar lateralization pattern) compared to ONM in CON, LAN, frontoparietal network (FPN), dorsal attention network (DAN), and default mode network (DMN), indicating preservation of youth-like lateralization patterns due to musical experience. Furthermore, in ONM, stronger left-lateralized and lower alignment-to-young of LI_intra in the somatomotor network (SMN) and DAN and LI_he in DMN correlated with better speech performance, indicating a functional compensation mechanism. In contrast, stronger right-lateralized LI_intra in FPN and DAN and higher alignment-to-young of LI_he in LAN correlated with better performance in OM, suggesting a functional preservation mechanism. These findings highlight the differential roles of functional preservation and compensation of lateralization in speech perception in noise among elderly individuals with and without musical expertise, offering insights into successful aging theories from the lens of functional lateralization and speech perception.

## Introduction

The global population is aging at an unprecedented rate, which presents significant challenges for families and society. Neurocognitive aging is characterized by multidimensional cognitive decline and prominent changes in brain structure and function [1–4]. Difficulty in understanding speech in noisy environments, commonly referred to as a “cocktail party problem”, is one of the salient concerns for older adults, even if their hearing is normal for their age. The Hemispheric Asymmetry Reduction in Older Adults (HAROLD) and the Scaffolding Theory of Aging and Cognition (STAC) models propose that the brain can adapt through compensatory mechanisms to counteract the effects of aging, resulting in increased fronto-parietal and bilateral recruitment and decreased lateralization [1, 5]. Furthermore, according to the revised STAC model [6], life-course factors, such as musical training, can enhance both the functional preservation and compensatory scaffolding processes, mitigating the aging effects. Indeed, empirical studies have demonstrated the benefits of both long-term and short-term musical training on speech perception in noisy environments for older adults [7–11], although such benefits are not consistently observed in young and middle-aged adults [12, 13]. However, it remains unclear whether and how functional preservation and compensation differentially support speech perception in noise in older adults with or without lifetime musical training experience. The answer to this question could help us develop effective and individualized interventions to improve listening skills and promote healthy aging in the elderly.

On one hand, older adults may engage in functional compensation to improve speech perception in noisy environments. The term ‘compensation’ refers to the cognition-enhancing recruitment of neural resources in response to relatively high cognitive demand. Compensation is achieved through three different forms: upregulation, selection, and reorganization by recruiting additional resources [14]. Previous studies have demonstrated the compensation mechanism as elevated activities in the cingulo-opercular network (CON, also known as the salience network) and frontoparietal network (FPN) in older adults, which support attention, working memory, and cognitive control [15–18]. Moreover, old adults upregulate task activity in frontal speech motor regions to compensate for speech perception in noise [19]. Functional compensation also involves the well-established age-related reduction of asymmetry, as proposed by the HAROLD model [3, 5]. Behavioral studies [20], as well as electroencephalography [21], positron emission tomography [22], near-infrared spectroscopy [23], resting-state and task functional magnetic resonance imaging (fMRI) studies [24, 25] provide evidence that brain networks become more bilateral and engage additional regions in the contralateral hemisphere to compensate for age-related neural declines.

Older musicians may also employ functional compensation to mitigate the effects of aging on speech perception. Compared to non-musicians, musicians exhibit enhanced auditory-motor integration during speech perception in noise, manifested by increased right lateralization of the dorsal stream’s key fiber [26], stronger activation of the right auditory cortex, and heightened functional connectivity between the right auditory and bilateral motor regions [27]. Furthermore, greater musical training-related plasticity has been observed in the right hemisphere of the brain, both structurally and functionally [28]. A recent study in older musicians has demonstrated increased recruitment of FPN regions and greater deactivation of the default mode network (DMN) regions as two means of functional compensation for speech in noise perception [11].

On the other hand, musical training is considered a ‘cognitive reserve’ that can delay age-related cognitive declines [7, 8]. The concepts of ‘reserve’ and ‘preservation’ (interchangeable terms in the literature) are consistent with evidence that adults who maintain stable cognitive performance as they age typically exhibit minimal age-related brain declines or changes [14]. Compared to older non-musicians, older musicians show enhanced central auditory processing functions and preserved cognitive abilities, including auditory attention and working memory, which may account for their comparable performance to young adults [9, 10, 29, 30]. Moreover, musical expertise in young adults has been found to help maintain right-lateralized ventral attention [31] and improve the neural specificity of speech representations in auditory and speech motor regions [27]. In older musicians, the preservation of youth-like neural specificity of speech representations in sensorimotor areas has been identified as a key mechanism for successful speech in noise perception [11]. Therefore, the benefit of musical expertise on speech perception in noisy environments among older adults is likely associated with the functional preservation of youth-like brain patterns as well.

**Fig. 1:**
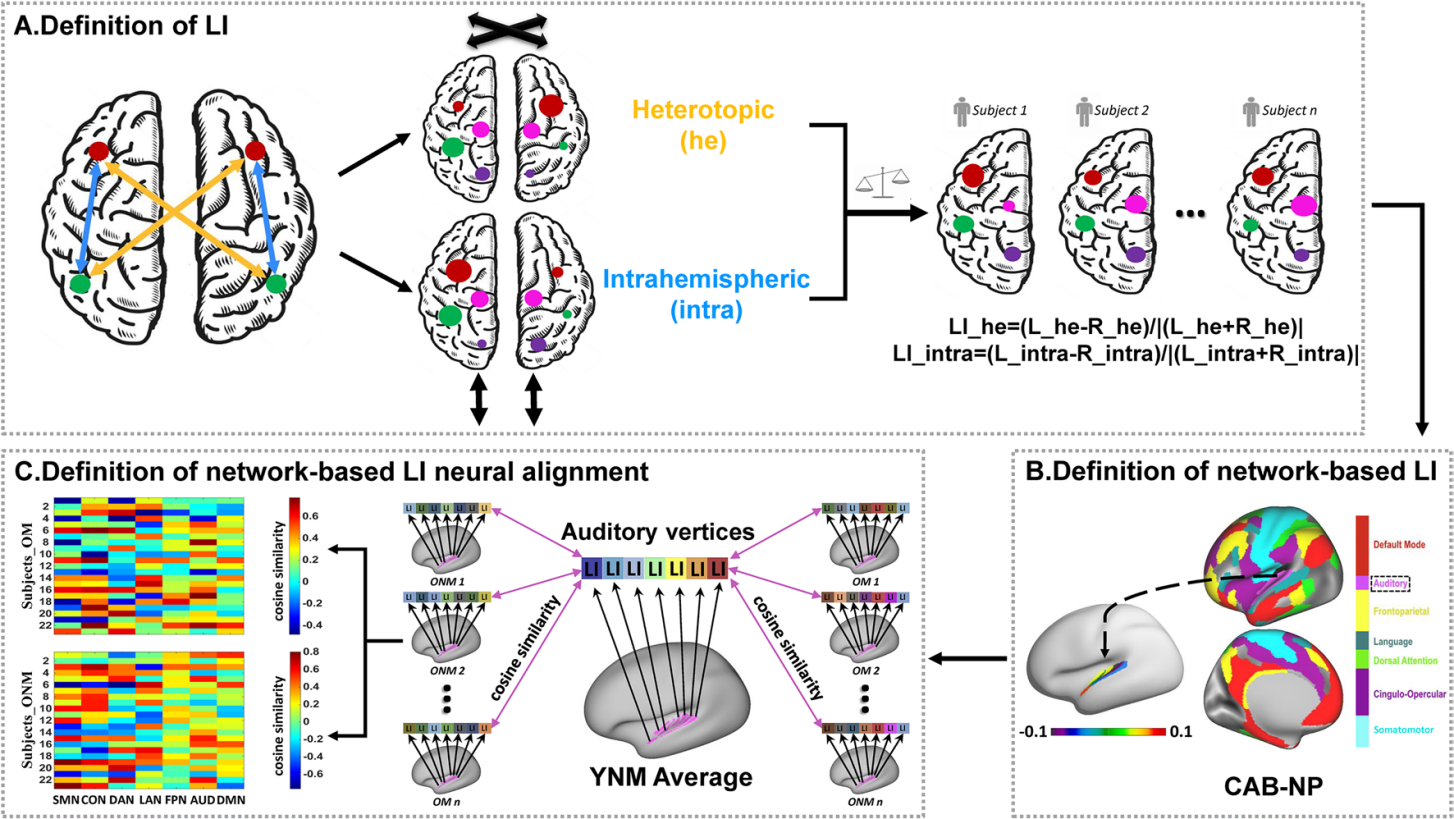
Workflow of analyses. **A** Definition of LI. We first defined two different types of functional connectivity (FC) among the whole brain, named interhemispheric heterotopic FC (yellow) and intrahemispheric FC (blue). For a specific surface vertex, the heterotopic (he) was defined as the sum of heterotopic FCs between this vertex and all the others in the opposite hemisphere except the homotopic one, whereas the intrahemispheric (intra) was defined as the sum of intrahemispheric FCs between this vertex and all the others within the same hemisphere. Then, the functional lateralization between each homotopic pair of surface vertices was quantified by a commonly used laterality index (LI): LI = (L−R)/|(L+R)|. Therefore, every subject would have two LI maps of he and intra. Larger positive values of LI_he and LI_intra imply stronger across-hemisphere interactions or within-hemisphere interactions in left-hemispheric vertices, respectively, whereas larger negative values indicate stronger interactions in right-hemispheric vertices. **B** Definition of network-based LI. Based on Cole-Anticevic Brain-wide Network Partition version 1.0 (CAB-NP v1.0), we acquired LIs of homotopic vertices belonging to seven task-relevant networks including somatomotor (SMN), cingulo-opercular (CON), dorsal attention (DAN), language (LAN), frontoparietal (FPN), auditory (AUD), and default-mode (DMN). **C** Definition of network-based LI neural alignment. Taking the AUD for example, we calculated cosine similarity between the group average of LI in young non-musicians (YNM) and LI in every older subject across all AUD vertices. By doing this, two cosine similarity matrices for seven networks of older non-musicians (ONM) and older musicians (OM) were obtained. Using the same approach, we also obtained the cosine similarity matrix of YNM.

In this resting-state fMRI (rs-fMRI) study, we aimed to investigate the specific brain mechanisms employed by older musicians (OM) and older non-musicians (ONM) to counteract age-related declines. Specifically, we tried to uncover how aging and lifelong musical training experience affect intrinsic functional lateralization and its relation with speech in noise perception ability. We utilized functional lateralization based on spontaneous brain activity at rest, as it reflects a fundamental organization characteristic of the brain’s intrinsic functional architecture and could be influenced by aging and musical experience [25, 32, 33]. Previous studies have shown that the degree of lateralization of resting-state functional connectivity (rsFC) predicts individuals’ language and visuospatial abilities, providing evidence for the association between functional lateralization of rsFC and human cognition [34–36]. In this study, we defined two types of lateralization indices (LIs) based on rsFC and compared them between older groups and young non-musicians (YNM): LI of intrahemispheric FC (LI_intra) which represents the left-right difference of functional connectivity strength within the same hemisphere, and LI of interhemispheric heterotopic FC (LI_he) which represents the left-right difference of functional connectivity strength across bilateral hemispheres [35–37]. Larger positive values of LI_intra and LI_he indicate stronger within-hemisphere and across-hemisphere interactions in the left hemisphere, respectively, whereas larger negative values imply stronger interactions in the right hemisphere. To further examine the functional lateralization of networks involved in functional compensation and preservation during the aging process with and without musical expertise, we performed correlations between speech perception thresholds and network-based LIs, as well as neural alignment-to-young. Neural alignment-to-young refers to the inter-subject spatial pattern similarity between the LI of each older subject and the group average of LI in YNM across the same network vertices (Fig. 1C). We hypothesized that ONM and OM would adopt different coping strategies against aging, resulting in different functional lateralization patterns and relationships between network-based LI and/or its neural alignment and speech perception thresholds. Specifically, ONM may exhibit functional compensation, with less similarity in lateralization to YNM indicating better performance, while OM may be more likely to reflect functional preservation, with greater similarity in lateralization to YNM associated with better performance.

## Materials and Methods

### Participants

Seventy-four healthy native Mandarin speakers with no history of psychiatric or neurological disorders participated in the experiment, including 24 YNM (23.13 ± 2.38 years, twelve females), 25 ONM (66.64 ± 3.40 years, sixteen females) and 25 OM (65.12 ± 4.06 years, eleven females). All participants had normal hearing in both ears with an average pure-tone threshold < 20 dB hearing level from 250 to 4000 Hz. OM started training before 23 years old (10.90 ± 4.56 years old) with at least 32 years of training (50.88 ± 8.75 years) and practiced consistently in recent three years (1–42 h per week, 12.70 ± 8.99 h per week). Non-musicians reported less than 2 years of musical training experience, which did not occur in the year before the experiment. To screen out participants with mild cognitive impairment, all older people passed the Montreal Cognitive Assessment (MoCA) of the Beijing version (≥ 26 scores) [38]. All participants reported their educational background and signed informed written consent approved by the ethical committee of the Institute of Psychology, Chinese Academy of Sciences. For more details, see Zhang *et al*. [11] which used the same participants.

### Behavioral Tests

#### Speech-in-noise (SIN)/Speech-in-Speech (SIS) Tasks

The speech in noise perception threshold was assessed using the SIN and SIS tasks in which syntactically correct but semantically meaningless speech sentences (for instance, “” “Some rules had translated my coat”) were embedded in a speech-spectrum noise (SIN task) or a two-talker speech masker (SIS task) at different signal-to-noise ratios (SNR = −12, −8, −4, 0, and 4 dB). The stimuli were presented binaurally through Sennheiser HD380 Pro headphones driven by a Dell desktop computer. The interaural time difference between the target sentence and the masker was manipulated to generate two perceived spatial relationships between the target and the masker: colocation and separation. Participants were asked to repeat the whole target sentence as best as they could immediately after the sentence was completed. A logistic psychometric function was employed in Matlab 2016b to fit each participant’s data for each masking and spatial relationship condition using the Levenberg–Marquardt method, and the SNR corresponding to 50% correct identification across two spatial relationships was used as the threshold ratio for the SIN and SIS tasks.

#### Digital Span Task

Auditory working memory was measured using the forward and backward Digit Span subtest of the Wechsler Adult Intelligence Scale of the Chinese version [39]. Participants were presented with a recording of a series of digits spoken by a young Chinese female. The number of digits increased from 3 to 12 in the forward part and from 2 to 10 in the backward part (two trials per length), while participants were asked to repeat the digits in a normal and reverse order, respectively. The task stopped if both trials for the same length were incorrect. The auditory working memory score was defined as the sum of the longest numbers participants could repeat in the forward and backward parts. Note that, raw scores instead of age-normed scores were used for analysis because the age difference was the research interest in the current study. Larger values indicate better performance.

#### Auditory Stroop Task

The Stroop test was adopted to evaluate inhibition control ability [40]. Participants were asked to listen to the Chinese words and then press the response key in synchronous condition (e.g., “high ()” in high pitch, the lexical meaning and pitch were always congruent), and in asynchronous condition (e.g., “high ()” in low pitch, the lexical meaning and pitch were always incongruent). Performance was indexed as the following: time of asynchronous condition minus time of synchronous condition. Larger values indicate poorer performance.

### Data Acquisition and Processing

Imaging data were collected using a 3 T MRI system (Siemens Magnetom Trio) with a 20-channel head coil. The high-resolution T1-weighted anatomical image was obtained using magnetization-prepared rapid acquisition with gradient echo (MPRAGE): repetition time (TR) = 2200 ms, echo time (TE) = 3.49 ms, a field of view (FOV) = 256 mm, flip angle (FA) = 8°, slice thickness = 1 mm, voxel size = 1 mm × 1 mm × 1 mm, 192 slices. Slowly fluctuating brain activity was measured using a multiband-accelerated echo-planar imaging (EPI) series with whole-brain coverage while subjects were instructed to rest still and quietly with their eyes fixated on a cross: TR = 640 ms, TE = 30 ms, FOV = 192 mm, FA = 25°, slice thickness = 3 mm, voxel size = 3 mm × 3 mm × 3 mm, 40 slices, multiband factor = 4. Each T1-weighted scan lasted 7 min 13 s and each resting-state scan lasted 8 min 6 s for a total of 750 consecutive whole-brain volumes.

Preprocessing was performed using fMRIPrep 20.2.5 [41]. The rs-fMRI data were preprocessed with slice-timing correction, motion correction, distortion correction, co-registration to structural data, normalization to Montreal Neurological Institute (MNI) space, and projection to the cortical surface. Functional time series were resampled to FreeSurfer’s fsaverage space, and the grayordinates files were generated. The eXtensible Connectivity Pipeline (XCP-D) [42] was then used to post-process the outputs of fMRIPrep. For each CIFTI run per subject, all data were subjected to demeaning, detrending, and nuisance regression. Volume with framewise displacement (FD) greater than 0.5 mm [43, 44] was flagged as outliers and excluded from nuisance regression. The nuisance regression pipeline was based on the empirical tests performed by Ciric *et al*. [45]. Specifically, six primary motion parameters were removed, along with their derivatives and the quadratics of all regressors (24 motion regressors in total). Physiological noise was modeled based on white matter and ventricle signals using aCompCor [46] within fMRIprep. Five component signals were used, as well as their derivatives and the quadratics of all physiological noise regressors (20 physiological noise regressors in total) [47]. Next, the residual time series from this regression were then band-pass filtered to retain signals within the 0.01 Hz –0.08 Hz frequency band. After smoothed with a Gaussian kernel size of 6 mm using FMRIB Software Library (FSL), processed functional time series were extracted from residual blood-oxygen-level dependent (BOLD) signals using Connectome Workbench [48]. For more details about post-processing, see the xcp_d website (https://xcp-d.readthedocs.io). In addition, we used FD to identify high movement frames in data (> 0.5 mm). By adopting “scrubbing” for each of these data points (head radius = 50 mm for computing FD, FD threshold = 0.5 mm for censoring), we excluded two OM with more than 20% data above the high motion cutoff (FD > 0.5). Moreover, one ONM for lacking complete behavioral data and one ONM who was left-handed were also excluded. Thus, seventy right-handed participants were included in the subsequent analysis.

### Definition of LI

To quantify rsFC at the surface level, we downsampled the original 32k time series into those with 10k vertices to accelerate further steps. Typically, the rsFC between two cortical surface vertices was computed as Pearson correlation (r) of two vertex-wise BOLD time series and then converted Fisher’s r-to-z transformation to improve the normality.

Based on the whole-brain rsFC matrix (z), we obtained intrahemispheric FC and interhemispheric heterotopic FC. The former represented the FC between two cortical surface vertices within the same hemisphere while the latter indicated the FC between two cortical surface vertices across different hemispheres, except the homotopic pairs. For a specific cortical surface vertex, the heterotopic (he) was defined as the sum of heterotopic FCs between this vertex and all the others in the opposite hemisphere except the homotopic one, whereas the intrahemispheric (intra) was defined as the sum of intrahemispheric FCs between this vertex and all the others within the same hemisphere. On these bases, for a pair of homotopic vertices, we further defined two different forms of functional lateralization, calculated as LI_he (equation 1) and LI_intra (equation 2).

1 $$LI\_he=\frac{\left({he}_{L}-h{e}_{R}\right)}{|{he}_{L}+{he}_{R}|}$$

2 $$LI\_intra=\frac{\left({intra}_{L}-intr{a}_{R}\right)}{|intr{a}_{L}+intr{a}_{R}|}$$

Larger positive values of LI_he and LI_intra imply stronger bilateral across-hemisphere interactions or ipsilateral within-hemisphere interactions in left-hemispheric vertices, whereas larger negative values indicate stronger interactions in right-hemispheric vertices. Finally, every participant would have two different LI maps of LI_he and LI_intra (Fig. 1A).

It is worth noting that our approach differs from a previous study [32], which calculated the left-right difference between the sum of average intra- and inter-hemispheric correlations in two hemispheres. Here, we defined two distinct LIs (LI_intra and LI_he) to separately quantify the left-right difference of intra- and inter-hemispheric correlations. This method allows for a more precise assessment of the lateralization of intra- and inter-hemispheric correlations, offering more nuanced insights into brain functional organization. Furthermore, the validation of these two LI measures has been confirmed by prior research [35–37].

### Definition of Network-Based LI and Its Neural Alignment

According to the Cole-Anticevic Brain-wide Network Partition version 1.0 (CAB-NP v1.0) [49], all cortical surface vertices were mapped into twelve networks. Since some homotopic pairs of vertices were labeled to different networks, we only chose the homotopic pairs of vertices whose left and right vertices both belonged to the same networks to calculate network-based LI *via* downsampled CAB-NP v1.0 from 32k to 10k. After averaging LIs within the same network, seven network-based LIs that are relevant to speech in noise tasks [50] and modulated by aging and musical training [51] were acquired, including somatomotor network (SMN), auditory network (AUD), language network (LAN), dorsal attention network (DAN), CON, FPN, and DMN (Fig. 1B). We further employed an inter-subject pattern correlation framework to examine spatial network-based LI similarities between every subject and the YNM average [11, 52]. For each network-based LI in every subject, we adopted an alignment-to-young measure by directly comparing the LI pattern of each subject and the mean LI pattern of YNM across the homotopic pairs of vertices belonging to the same network, using cosine similarity. By doing this, three cosine similarity matrices of YNM, ONM, and OM for seven networks were obtained (Fig. 1C). Higher neural alignment implies a more similar functional lateralization pattern to YNM, whereas lower neural alignment indicates a less similar functional lateralization pattern to YNM. All analyses above were performed on both LI_he and LI_intra.

### Statistical Analysis

To highlight the differences in LI_he and LI_intra between the three groups, we presented the averaged global maps of LIs in YNM, ONM, and OM, respectively. Next, one-sample t-tests were performed on seven network-based LIs separately to identify whether this functional network was lateralized (leftward/rightward) or symmetric for each group. One-way analyses of variance (ANOVAs) for network-based LIs were adopted to compare the group differences. As for similarity differences of network-based LI, one-way ANOVAs and Jonckheere-Terpstra tests were used to validate whether a trend (YNM > OM > ONM) existed in network-based LI neural alignments in three groups.

Since we intended to explore the relationship between functional lateralization and behavior, partial correlations were used to evaluate the relation between network-based LIs as well as their neural alignments and speech in noise performances in two older groups. considering age, gender, education, mean framewise displacement (mFD), mean global FC (mean of rsFCs between any two cortical surface vertices in the whole brain), hearing level, digit span, Stroop, and MoCA which were related to speech perception in noise as confounding variables.

All the results in our analyses were considered to be significant with values below 0.05 after false discovery rate (FDR) or Games-Howell corrections (one-way ANOVA with heterogeneity of variance). The brain maps were projected to surfaces by SurfStat package (http://www.math.mcgill.ca/keith/surfstat) and Connectome Workbench platform (http://www.humanconnectome.org/software/connectome-workbench.html).

## Results

### Musical Expertise Counteracts Age-Related Decline of Speech Perception in Noisy Environments

Twenty-four YNM, 23 OM, and 23 ONM completed the experiment. The two older groups exhibited no significant difference in age, gender, MoCA, and hearing level, but a significant difference in education (*t*_44_ = 3.769, Cohen‘s *d* = 1.112, *P <* 0.001). Among three groups, separate one-way ANOVA showed a significant main effect of group on speech perception threshold with noise masking (SIN: Welch *F*(2, 38.091) = 25.070, *η*_*p*_^2^ = 0.526, *P <* 0.001), speech perception threshold with speech masking (SIS: Welch *F*(2, 38.920) = 28.217, *η*_*p*_^2^ = 0.568, *P <* 0.001), and auditory digit span (*F*(2, 67) = 28.749, *η*_*p*_^2^ = 0.462, *P <* 0.001). Post hoc analysis revealed that ONM showed significantly worse performance on each task above than OM and YNM [all FDR-corrected or Games-Howell-corrected *P <* 0.001], but no significant difference was found between OM and YNM, suggesting that musical expertise can offset older adults’ decline of speech perception in noisy environments. For more details, see Table 1.

**Table 1:** The group mean ± standard deviation values and statistics of demographic and behavioral data in each group

	YNM	OM	ONM	*t/χ2/Welch F/F* (*P*)
Age (year)	23.13 ± 2.38	64.61 ± 3.76	66.70 ± 3.34	1.207^a^ (0.053)
Gender (F/M)	12/12	9/14	14/9	2.174^b^ (0.337)
Education (year)	16.50 ± 1.67	13.15 ± 3.06	9.89 ± 2.80	− 3.769^a^ (< 0.001)
MoCA (score)	NA	27.91 ± 1.24	27.65 ± 1.37	− 0.677^a^ (0.502)
Hearing level (dB HL)	0.71 ± 3.32	12.33 ± 5.42	12.57 ± 4.01	0.170^a^ (0.866)
Speech-in-noise perception (dB)	− 3.99 ± 0.59	− 3.61 ± 0.97	− 1.04 ± 1.90	25.070^c^ (< 0.001)
Speech-in-speech perception (dB)	− 5.43 ± 1.00	− 4.97 ± 1.51	− 0.31 ± 3.09	28.217^c^ (< 0.001)
Digit span (number)	16.71 ± 2.71	15.09 ± 2.27	11.57 ± 2.06	28.749^d^ (< 0.001)
Stroop (second)	0.18±0.12	0.18 ± 0.10	0.23 ± 0.19	0.974^d^ (0.383)
Age of training onset (age)	NA	11.17 ± 4.66	NA	
Years of training (year)	NA	49.84 ± 8.26	NA	

*YNM* young non-musicians, *ONM* older non-musicians, *OM* older musicians.

MoCA, Montreal Cognitive Assessment; NA, data were not collected.

One-way analyses of variance, independent two-sample t-tests, and Chi-square tests were used for examining the group differences.

^a^Independent two-sample t-test (for age, education, MoCA, and hearing level between ONM and OM).

^b^Chi-square test.

^c^One-way analysis of variance with *Welch F* value.

^d^One-way analysis of variance with *F* value.

### Musical Expertise is Associated with Less Age-Related Hemispheric Lateralization Reduction

**Fig. 2:**
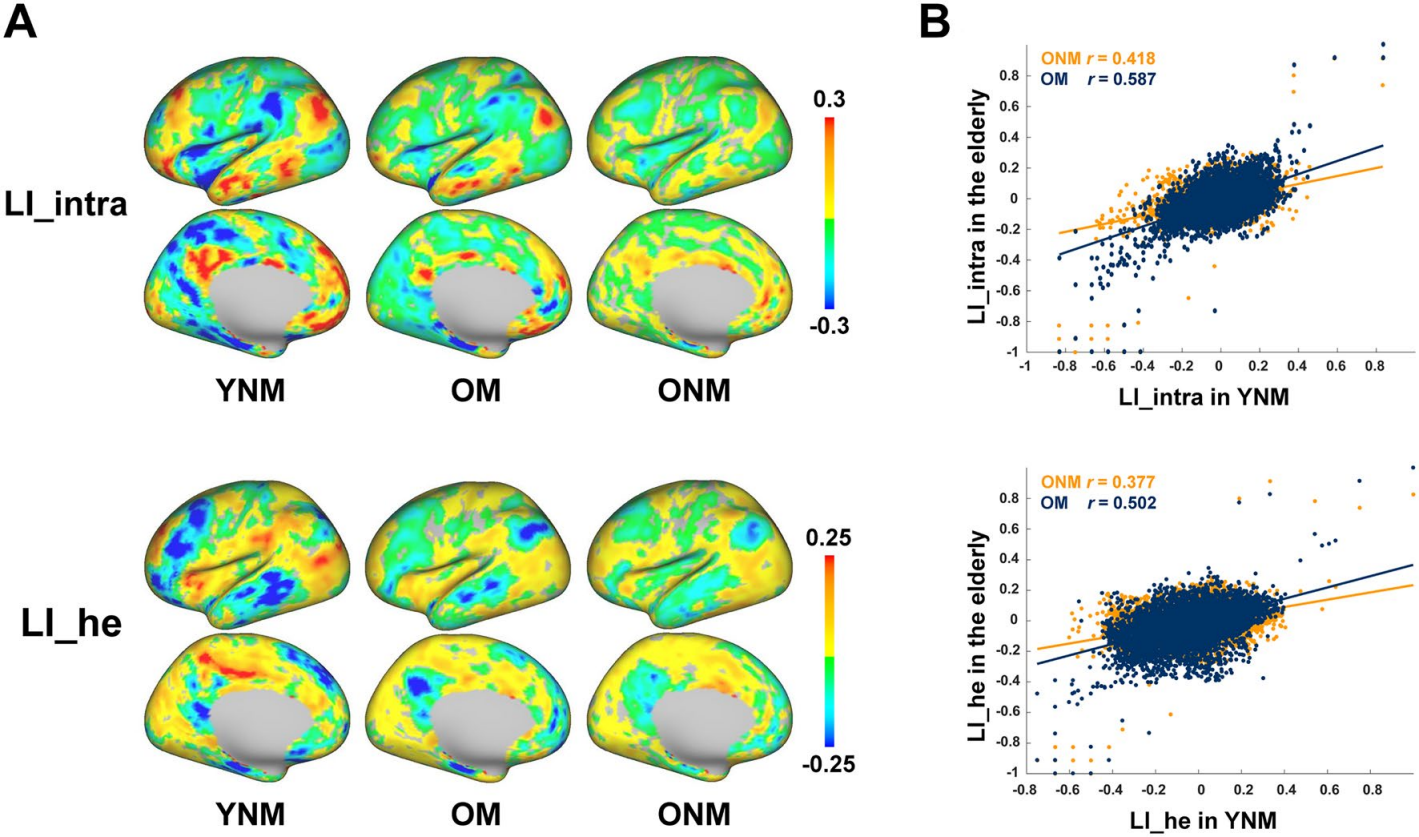
Vertex-based LI in three groups. **A** Group averaged global maps of vertex-based LI_intra and LI_he in YNM, OM, and ONM. **B** Correlations between group-averaged LIs in YNM and two older groups across vertices, with OM showing significantly higher correlations than ONM (OM, blue; ONM, yellow).

To reveal how aging and musical expertise influence brain functional lateralization, we examined the difference in LIs between YNM, ONM, and OM. We first calculated global LI maps of LI_intra and LI_he at the vertex level for each group. As shown in Fig. 2A, YNM presented left-lateralized LI_intra in language-related and DMN areas and right-lateralized LI_intra in CON areas, which are consistent with previously observed patterns [35]. However, the results of LI_intra in OM and LI_he in all three groups have not been reported before. Generally speaking, ONM showed a more symmetrical and substantially diminished lateralization pattern compared to YNM, while OM maintained a more similar lateralization pattern to YNM. These differences in global lateralization pattern were further verified by the correlations between group-averaged LIs in YNM and those in two older groups across all vertices (Fig. 2B), with OM and YNM having higher *r* values than ONM and YNM (LI_intra: ONM, *r* = 0.418, *P <* 0.001, OM, *r* = 0.587, *P <* 0.001, ONM vs. OM, *z* = 15.608, *P <* 0.001; LI_he: ONM, *r* = 0.377, *P <* 0.001, OM, *r* = 0.502, *P <* 0.001, ONM vs. OM, *z* = 10.606, *P <* 0.001).

**Fig. 3:**
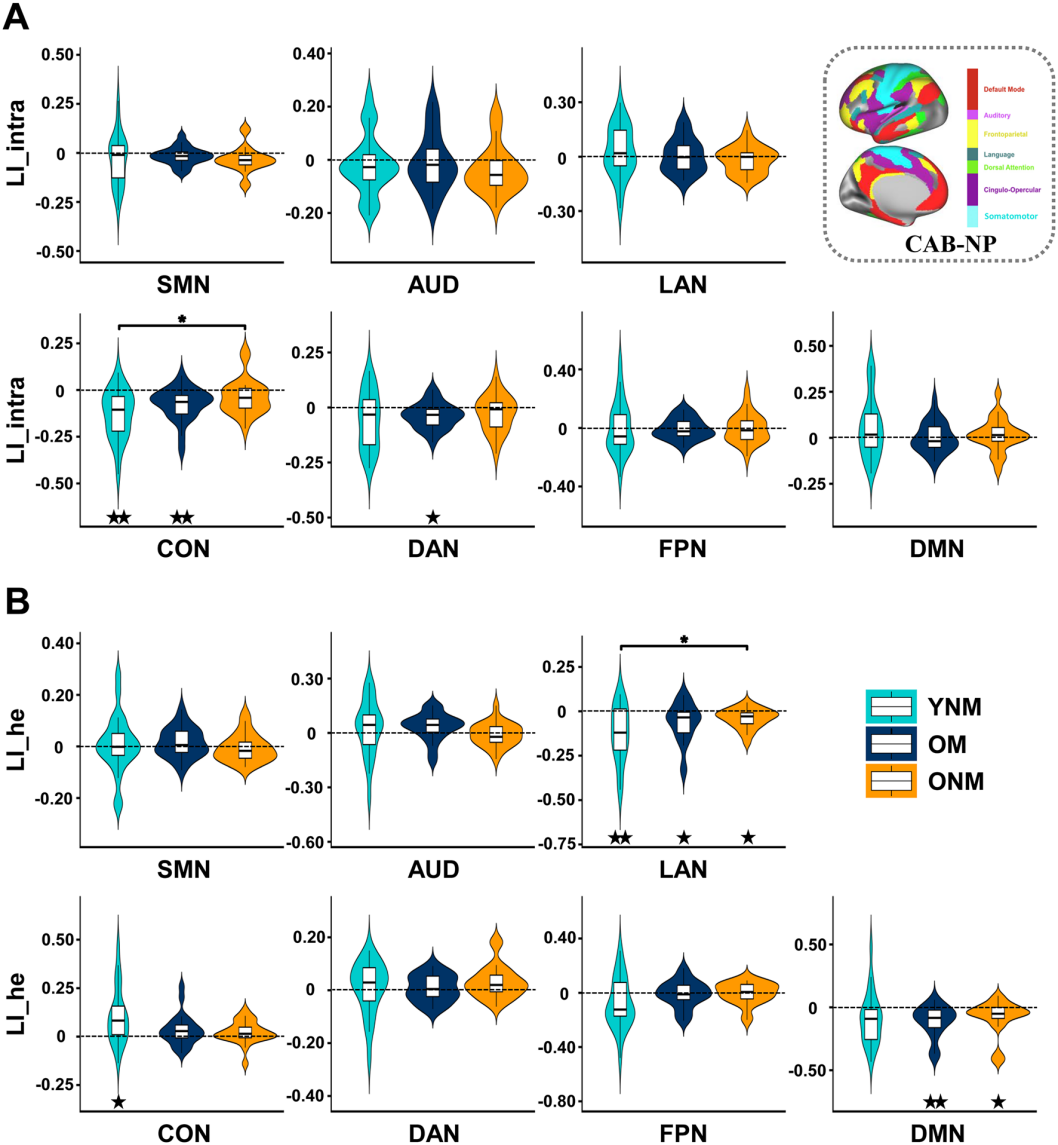
Group comparisons of network-based LI. LI_intra (**A**) and LI_he (**B**) were compared among YNM, OM, and ONM in each of the seven networks. * FDR-corrected or Games-Howell-corrected *P* < 0.05 by post hoc tests after one-way analysis of variances. ^★^FDR-corrected *P* < 0.05, ^★★^FDR-corrected *P* < 0.01 by one-sample t-tests. All dashed lines indicate zero (no functional lateralization).

To confirm our findings at the network level, we calculated seven network-based LI_intra and LI_he by averaging the LIs from the same network based on global LI maps. One-way ANOVAs revealed a group difference on LI_intra of CON (*F*(2, 67) = 4.706, *η*_*p*_^2^ = 0.123, uncorrected *P =* 0.012) and LI_he of LAN (Welch *F*(2, 38.241) = 3.313, *η*_*p*_^2^ = 0.093, uncorrected *P =* 0.047). Post hoc tests demonstrated that ONM showed a significantly larger LI (close to zero) than YNM (LI_intra of CON: FDR-corrected *P =* 0.021; LI_he of LAN: Games-Howell-corrected *P =* 0.044) but no difference was discovered between OM and two non-musician groups (Fig. 3). Further one-sample t-tests found a significantly right-lateralized LI_intra of CON in YNM and OM (both *t* < − 4.010, Cohen‘s *d* < − 0.855, FDR-corrected *P <* 0.01) but not in ONM, and a significantly right-lateralized LI_he of LAN in three groups (all *t* < − 2.773, Cohen‘s *d* < − 0.578, FDR-corrected *P <* 0.05). These results supported the HAROLD model and suggested that aging was associated with reduced hemispheric asymmetry especially in CON and LAN, while musical expertise counteracted such changes in functional lateralization, making OM more alike YNM.

### Musical Expertise is Associated with Enhanced Neural Alignment-to-Young of Lateralization in Older Adults

**Fig. 4:**
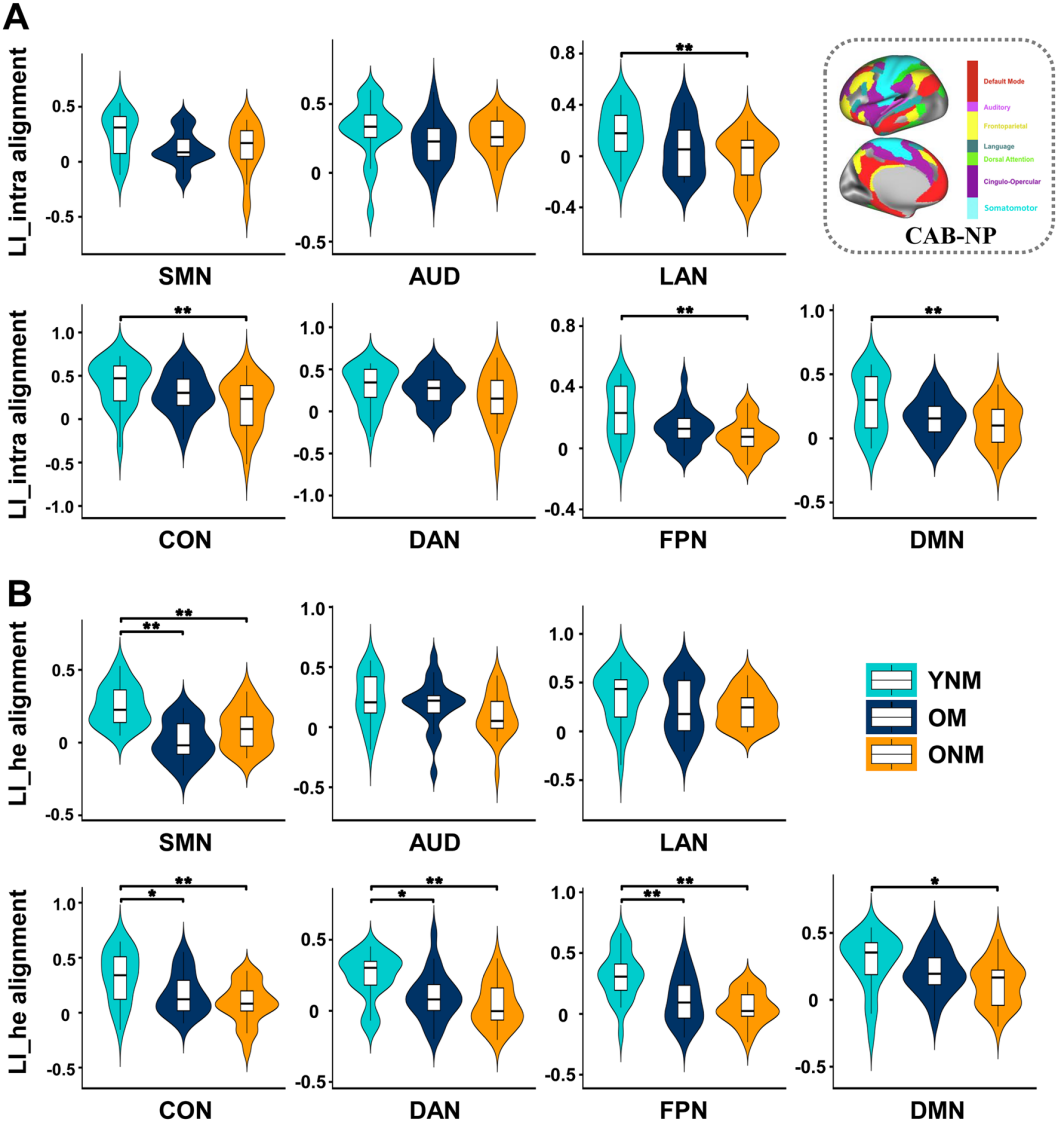
Group comparisons of neural alignment-to-young of network-based LI. Neural alignment-to-young of LI_intra (**A**) and LI_he (**B**) were compared among YNM, OM, and ONM in each of the seven networks. *FDR-corrected *P* < 0.05, **FDR-corrected or Games-Howell-corrected *P* < 0.01 by post hoc tests after one-way analysis of variances.

As shown above, OM had a more similar lateralization pattern as YNM. To directly verify this phenomenon, we adopted the neural alignment-to-young measure of network-based LI. One-way ANOVAs on the neural alignment of LI_intra (Fig. 4A) demonstrated significant group differences in several networks including LAN, CON, FPN, and DMN (all *F/Welch F* > 5.082, *η*_*p*_^2^ > 0.135, all FDR-corrected *P <* 0.05). Post hoc tests revealed that ONM showed significantly lower alignment-to-young than YNM in all four networks (all FDR- or Games-Howell-corrected *P <* 0.01), but no significant difference was discovered between OM and YNM. In parallel, significant group differences were found in neural alignment of LI_he in SMN, CON, DAN, FPN, and DMN (all *F* > 4.912, *η*_*p*_^2^ > 0.128, all FDR-corrected *P <* 0.01, Fig. 4B). Post hoc tests showed significantly lower alignment in both ONM and OM than in YNM in SMN, CON, DAN and FPN (all FDR-corrected *P <* 0.05). For DMN, a significant difference was found only between YNM and ONM (FDR-corrected *P =* 0.014), but not between YNM and OM. For networks with significant group differences in neural alignment, we further used the Jonckheere-Terpstra (J–T) tests to validate whether a trend of neural alignments (YNM > OM > ONM) existed in three groups. Results showed a significant trend (YNM > OM > ONM) in the neural alignment of LI_intra in LAN, CON, FPN, DMN (all J–T statistics < − 1.666, all FDR-corrected *P <* 0.05) and LI_he in CON, DAN, FPN, and DMN (all J–T statistics < − 1.741, all FDR-corrected *P <* 0.05). Together, we confirmed that OM preserved youth-like lateralization patterns in networks including LAN, CON, DAN, FPN, and DMN. Additionally, no significant correlation was found between network-based LI or its alignment-to-young and years of musical training or the age of training onset in OM, except that the LI_he (*r* = 0.430, uncorrected *P =* 0.040) and its neural alignment (*r* = − 0.502, uncorrected *P =* 0.015) in SMN were correlated with the age of training onset, indicating stronger benefit for early learners.

### Functional Lateralization Differentially Contributes to Speech Perception in Noisy Environments in Older Musicians and Older Non-musicians

Since OM and ONM presented distinct functional lateralization patterns compared to YNM, we then determined which networks exhibited functional compensation and which ones demonstrated functional preservation by investigating the relationship between functional lateralization as well as its neural alignment and speech in noise perception performance in two older groups. Notably, hearing level, working memory, and inhibitory control were related to speech perception in noise, and significant group differences were found in education, mFD (*t*_44_ = − 2.147, Cohen’s *d* = − 0.633, *P <* 0.05). We thus regressed out age, gender, education, hearing level, MoCA, digit span, Stroop, mFD and mean global FC (*t*_44_ = − 1.055, *P =* 0.297), and conducted partial correlations of network-based LI and its neural alignment with speech perception threshold in the two older groups, respectively.

**Fig. 5.**
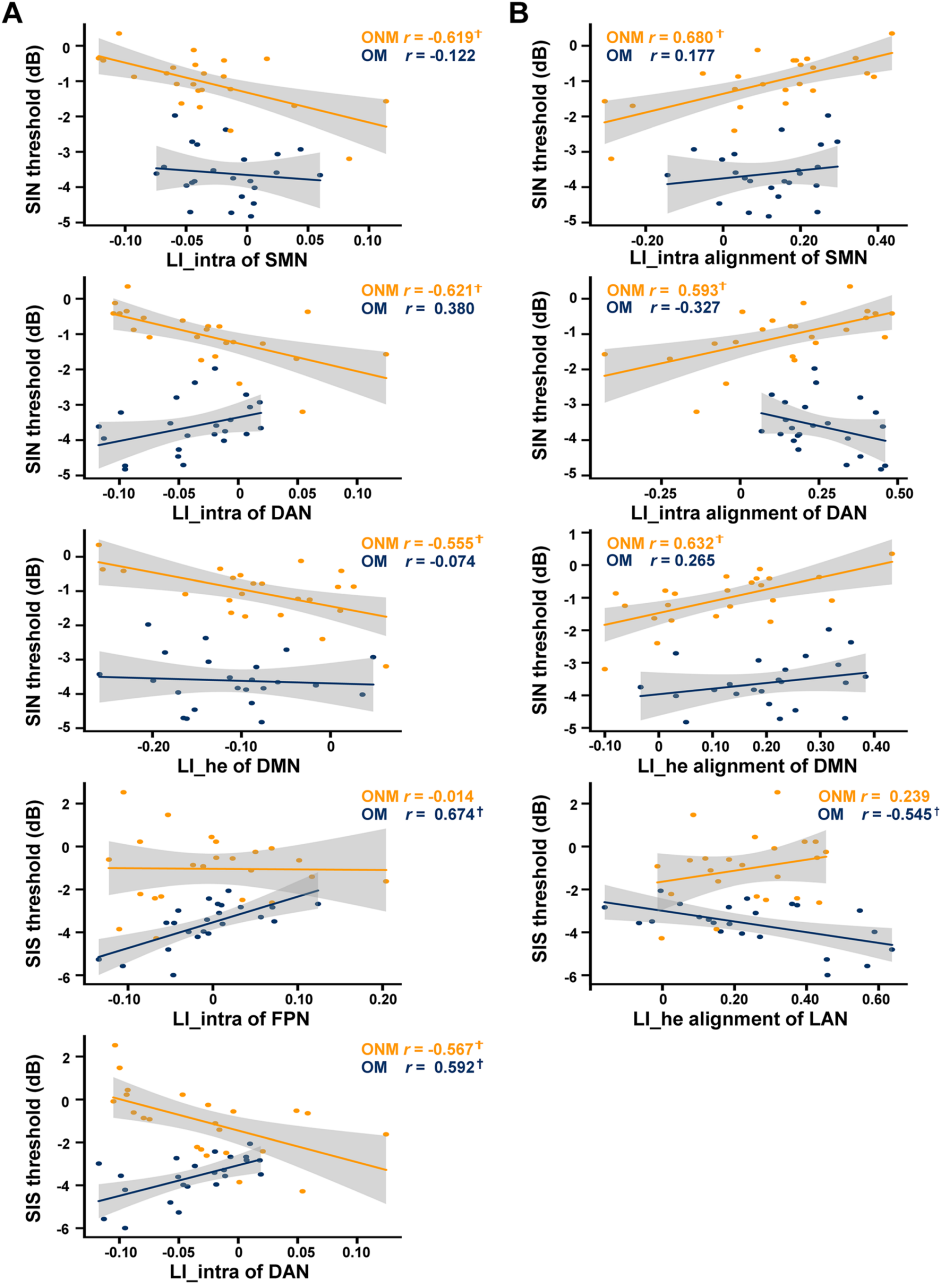
Correlations of network-based LI and its neural alignment with behaviors.** A** Partial correlation between network-based LI and speech-in-noise (SIN) threshold or speech-in-speech (SIS) threshold in OM and ONM, after controlling for gender, education, hearing level, digit span, Stroop, MoCA, mean framewise displacement and mean global functional connectivity. **B** Similar partial correlations between network-based LI alignment and SIN or SIS threshold. ^†^ uncorrected *P* < 0.05.

As shown in Fig. 5A, lower SIN or SIS threshold (representing better performance) was correlated with more left-lateralized LI_intra in SMN (SIN: *r* = − 0.619, uncorrected *P =* 0.018) and DAN (SIN: *r* = − 0.621, uncorrected *P =* 0.018; SIS: *r* = − 0.567, uncorrected *P =* 0.035), as well as more left-lateralized LI_he in DMN (SIN: *r* = − 0.555, uncorrected *P =* 0.040) in ONM. In contrast, in OM, a lower SIS threshold was correlated with more right-lateralized LI_intra in FPN (*r* = 0.674, uncorrected *P =* 0.008, FDR-corrected *P =* 0.058) and DAN (*r* = 0.592, uncorrected *P =* 0.026). Therefore, the two older groups displayed quite opposite relationships between functional lateralization and speech perception threshold.

Furthermore, we investigated whether superior speech in noise perception ability in older adults with or without lifetime musical experience was linked to a functional lateralization pattern similar to that of young adults or the opposite. Using partial correlations between network-based LI neural alignments and speech perception thresholds, we found that in ONM, lower neural alignment of LI_intra in SMN (*r* = 0.680, uncorrected *P* = 0.007, FDR-corrected *P =* 0.052) and DAN (*r* = 0.593, uncorrected *P* = 0.025), as well as lower alignment of LI_he in DMN (*r* = 0.632, uncorrected *P =* 0.015) were correlated with lower SIN threshold (Fig. 5B). This suggests that older non-musicians who demonstrated less similarity to young adults in their lateralization pattern exhibited better speech perception in noisy conditions, implying the importance of functional compensation for lateralization. Conversely, in OM, higher neural alignment of LI_he in LAN (*r* = − 0.545, uncorrected *P =* 0.044) was correlated with a lower SIS threshold (Fig. 5B). This suggests that older musicians who displayed a lateralization pattern more similar to young adults showed improved speech perception in a noisy environment, indicating a pattern of functional preservation of lateralization.

## Discussion

Through an unexplored lens of intrinsic brain functional lateralization, our study provides evidence that long-term musical training mitigates age-related decline in speech perception in noisy environments. This effect is likely achieved through the preservation of youthful functional lateralization patterns. Conversely, older adults lacking musical training experience appear to rely on a stronger scaffolding of compensatory hemisphere with less similar functional lateralization patterns to young adults in order to maintain speech perception performance. In our study, age-related decline in perceiving speech sentences in “cocktail party” scenarios (with either speech or noise masker) was found only in ONM but not in OM. In parallel with behavioral findings, right-lateralized intrahemispheric FC in CON and interhemispheric heterotopic FC in LAN in YNM were transitioned towards a bilaterally symmetric pattern in ONM but not in OM. In addition, ONM was less similar to the YNM template than YNM in the functional lateralization of most networks, while OM maintained a youthful lateralization pattern in networks including LAN, CON, DAN, FPN, and DMN. Moreover, functional lateralization contributed to speech in noise perception differently for OM and ONM. In ONM, better performance was associated with lower neural alignment-to-young (i.e., a less similar lateralization pattern to YNM) and a more left-lateralized pattern, suggesting a functional compensation mechanism. Conversely, in OM, better performance was associated with higher neural alignment-to-young (i.e., a more similar lateralization pattern to YNM) and a more right-lateralized pattern, suggesting a functional preservation mechanism. Therefore, successful aging in speech perception can be achieved through opposite functional lateralization changes in older adults with and without musical training.

### Functional Compensation in Older Non-musicians

It is widely accepted that the aging brain responds to anatomical and physiological changes by reorganizing its function [5]. Previous studies have shown that older adults show a more bilateral pattern of prefrontal activity during verbal recall, which has been interpreted as functional compensation [5]. Resting-state fMRI studies also reported a decrease in lateralization with aging in several networks, such as sensorimotor, attentional, and frontal networks [25, 53]. These findings convergently support the notion that increased bilateral recruitment and decreased lateralization serve as compensatory scaffolding mechanisms for age-related neural decline [1].

Our study further substantiates these findings by revealing significant changes from lateralization in YNM to bilateralization in ONM, especially in the CON and LAN networks. Although asymmetry reduction in older adults is commonly interpreted as a compensatory mechanism [5, 6], the diminished lateralization in CON and LAN did not directly influence speech in noise perception in ONM in the present study. This suggests neural inefficiency [54] or neural dedifferentiation [2] rather than compensation. Instead, when confronted with speech-in-noise tasks that require high cognitive demand, older adults necessitate additional neural recruitment from alternative pathways that compensate for their performance, which younger individuals do not typically utilize. Specifically, ONM performed better when their functional lateralization pattern was dissimilar to that of YNM, presenting a more left-lateralized pattern in other task-relevant networks such as SMN, DAN, and DMN as a result of functional compensation by reorganization. Previous task fMRI studies have demonstrated the compensatory role of left speech motor areas [19] and bilateral sensorimotor regions [11] for poor speech encoding under adverse listening conditions in ONM. Our resting-state functional lateralization findings further support the hypothesis that sensorimotor integration serves as a compensatory scaffolding mechanism for speech perception in the aging group, showing that high-performing ONM had a more bilateral and even left-lateralized SMN, deviating from the pattern observed in young adults. In addition, consistent with the posterior-anterior shift in aging and the decline-compensation hypothesis [22, 55], ONM also recruits more frontal areas to compensate for declined sensory functions when performing speech in noise tasks [17–19]. Since the ability to track and understand speech amid competing sound sources is supported by higher-level cognitive processes such as selective attention [56–58], the functional lateralization pattern of DAN and its relationship with the speech in noise perception in high-performing ONM might represent another compensatory mechanism for greater listening effort. Furthermore, successful speech perception in noise requires inhibiting task-unrelated long-term memory supported by DMN. Older adults have been reported to exhibit deficits in cognitive control and resource reallocation to task-related regions, indicating that failure to inhibit DMN in the elderly is detrimental to task performance [59, 60]. In high-performing ONM, we detected a less similar functional lateralization pattern to that of YNM, displaying more bilateral and even leftward lateralization of DMN. This finding supports the compensation theory that higher-order cognitive networks may become more bilateral and even display an opposite lateralization pattern to compensate for sensory declines. In summary, older adults without musical training experience may rely on functional compensation by reorganizing the functional lateralization across multiple networks involved in sensorimotor integration, attention, and inhibition of long-term memory to maintain speech perception in noisy environments. These results highlight the role of reorganization as a form of compensation among the three mechanisms proposed (upregulation, selection, and reorganization) [14].

### Functional Preservation in Older Musicians

Various factors, such as experience, genetics, and environment, influence the aging process and cognitive function [6]. Life-course variables, which encompass an individual’s cumulative experiences and states from birth to death [61], would impact the structure and function of the aging brain [6]. Long-term musical training experience is one of those variables that have been found to improve auditory and cognitive functions, particularly for the aging population in adverse listening environments [7, 10]. Here, functional lateralization of CON and LAN in YNM was weakened in ONM but well preserved in OM. Neural alignment analysis further revealed more evident discrepancies between YNM and ONM than between YNM and OM in LAN, CON, DAN, FPN, and DMN, demonstrating that musical training experience helps older adults preserve a youth-like functional lateralization pattern in networks associated with language processing, attentional and cognitive control. These widespread youthful lateralization patterns are consistent with previous studies [31, 35], which have shown that musical training has an age-decelerating effect on the brain [7, 10, 11]. Compared to non-musicians, musicians’ predicted brain age was younger than their chronological age using a machine-learning algorithm [62].

Moreover, this functional preservation of lateralization supported speech in noise perception in OM. A recent task fMRI study found that OM demonstrated better speech-in-noise perception performance through functional preservation by maintaining similar speech representation patterns as young adults [11]. In our resting-state fMRI study, high-performing OM showed stronger right lateralization in DAN and FPN, which contrasted with the patterns observed in ONM. These findings align with previous studies revealing a bilateral dorsal attention system [63] and increased recruitment of the right hemisphere in speech processing in musicians [26, 27]. As speech in noise perception engages the allocation of attentional resources and inhibitory control [64], that facilitate target signal segregation from background noise [65], the enhancement of these higher-level cognitive processes in DAN and FPN has been correlated with improved speech in noise perception in musicians [10, 29, 66, 67]. According to the OPERA (overlap, precision, emotion, repetition, attention) hypothesis, musicians’ advantage in speech processing is driven by the anatomical overlap in the brain networks that process music and speech [68]. Given the partial overlap between neural circuits dedicated to music and language [69], long-term musical training may continuously influence language networks and help preserve the youthful lateralization pattern in LAN among older musicians. Our study supports this notion, as we found that high-performing OM showed a similar lateralization pattern with higher alignment-to-young in LAN. In summary, the functional lateralization patterns in DAN, FPN, and LAN potentially enable older musicians to maintain cognitive abilities associated with selective attention, inhibitory control, and language, contributing to better speech perception performance. Since successful preservation can explain cross-sectional findings that the brains of high-performing older adults exhibit similar anatomy and physiology to young brains, while the brains of low-performing older adults differ from young brains [70], our results indicate a functional preservation mechanism in older musicians contrary to the functional compensation mechanism in older non-musicians. Notably, ‘preservation’ refers to the maintenance of neural resources, which entails ongoing repair and replenishment of the brain in response to declines, whereas ‘reserve’ is defined as a cumulative improvement of neural resources that mitigates aging-related neural declines [14]. Although both concepts involve enhancing current neural resources, the reserve is about augmenting neural resources beyond their current level, whereas preservation is about returning them to their former higher level. Therefore, despite musical training being considered a ‘cognitive reserve’, our findings align more closely with the mechanism of functional preservation rather than the functional reserve.

### Limitations

Due to the rarity of older musicians, especially those eligible for strict inclusion criteria and willing to participate in our fMRI study, we encountered limitations related to sample size. As a result, some of our results cannot be corrected for multiple comparisons, although the correlations were substantial enough to support certain findings. Additionally, we only found a significant difference in neural alignment of network-based LI between ONM and YNM, but not between OM and YNM nor between OM and ONM. However, we did observe a trend in LI similarity (YNM > OM > ONM) across several networks. These results provide moderate evidence suggesting that older musicians have a functional lateralization pattern more similar to young adults than older non-musicians. Conducting future research with a larger sample size will help to directly verify the difference between older musicians and older non-musicians. While resting-state functional connectivity offers valuable insights into the intrinsic functional organization of the brain, its sole predictive power as a phenotype for specific cognitive abilities, such as speech perception in older adults, may be limited. Future studies that integrate other factors, such as structural brain changes, cognitive reserve, and individual variability in neural plasticity, could provide a more comprehensive understanding of the determinants of speech perception abilities in older adults. By considering these additional factors, researchers can contribute to a richer and more nuanced exploration of this field.

To sum up, our study offers a previously unidentified perspective on the resting-state functional lateralization and its relationship with speech perception in noisy environments. We discovered that older non-musicians showed a reduction in hemispheric lateralization compared to young adults, while older musicians maintained a youthful pattern of lateralization. Furthermore, older non-musicians relied on compensatory networks with a more bilateral and even left-lateralized pattern to sustain their speech in noise perception, while older musicians depended on preserving a youth-like functional lateralization with a more right-lateralized pattern. These distinct coping strategies against aging in older non-musicians and older musicians deepen our understanding of functional compensation and functional preservation in aging theories and provide insights for individualized training interventions.

## Data Availability

The data that support the findings of this study are available at https://osf.io/2wxhv/.
